# The dysplastic trochlear sulcus due to the insufficient patellar stress in growing rats

**DOI:** 10.1186/s12891-019-2802-y

**Published:** 2019-09-05

**Authors:** Guangmin Yang, Faquan Li, Jiangfeng Lu, Yingzhen Niu, Yike Dai, Lixiong Zuo, Gengshuang Tian, Fei Wang

**Affiliations:** Department of joint surgery, Third Hospital of Hebei Medical University, Shijiazhuang, 050051 Hebei China

**Keywords:** Knee, Patellofemoral joint, Trochlear dysplasia, Rats, Biomechanical stress

## Abstract

**Background:**

Developmental factors were assumed to be the key factors that influenced the morphology of femoral trochlea. This study investigated the effects of insufficient patellar stress after birth on the morphological development of the femoral trochlea. Effects of insufficient patellar stress on femoral trochlea were investigated using surgical induced patellectomy and patellar dislocation in growing rat model.

**Methods:**

In this study, two experimental groups and one sham group (SG) were established. Thirty-six Wistar rats (female, 28 days of age) were randomly assigned to three groups. The patellectomy group (PG), rats underwent the patellectomy in this group. The dislocation group (DG), rats underwent the surgery-induced patellar dislocation. Histological staining (Safranin-O and fast green), Micro-computed tomographic (Micro-CT) analysis in two experimental endpoints (3, 12 weeks postoperatively) were selected to evaluate morphological changes of the femoral trochlea.

**Results:**

Articular cartilage on the trochlear sulcus was remodeled at 3 weeks after the surgery, and degenerated at 12 weeks through the histological staining. The femoral trochlear angle (FTA) did not show a significant difference at 3 week between the experimental groups and the sham group (PG vs SG *P* = 0.38, DG vs SG *p* = 0.05), but the FTA was significantly increased in experimental groups at 12 weeks(PG vs SG *P* = 0.001, DG vs SG *p* = 0.005). The Bone volume density (BV/TV), Trabecular number (Tb.N) under the trochlea groove were significantly reduced at 3 weeks postoperatively in the experimental groups (PG vs SG *p* = 0.001, DG vs SG *p* = 0.002). No significant difference was found in BV/TV and Tb. N among the three groups at 12 weeks postoperatively.

**Conclusion:**

Surgical induced patellectomy and patellar dislocation leads to the dysplastic trochlear sulcus in growing rats. Besides the bone morphology of trochlear sulcus, the articular cartilage and subchondral trabecula under the trochlear sulcus were remodeled early stage after the surgery.

## Background

Previous studies had reported the high incidence of patellar dislocation (up to 29 to 43 of 100,000 people) in the teenagers [1, 2]. Trochlear dysplasia (TD) is a major predisposing factor for patellar dislocation. Researches by Dejour and Fulkerson et al. had reported that trochlear dysplasia existed in 96% of patients with a history of patellar dislocation [3]. The prominent features of trochlear dysplasia are the flat trochlear sulcus or irregular trochlear facets. In 1964, Brattstrom first reported the potential association with trochlear dysplasia and the occurrence of patellar instability [4]. In current years, researches had proved that the development of trochlear dysplasia might be related to the following risk factors, such as patella Alta, excessive femoral anteversion, and higher TT-TG distance [5–10].

Trochlear dysplasia and patellar dislocation, which come first are still hard to determine in the current researches [11]. In a retrospective study, Parikh et al. found that the dysplastic femoral trochlea has already formed in childhood using radiological measurements [5]. Øye et al. found children with the breech presentation has a high-risk factor of trochlear dysplasia and patellar dislocation and supposed the morphology of the trochlear groove was initially formed in the prenatal stage when the osseous trochlea began its development [12]. Experimental researches based on the animal model had also supported the role of acquired factors on the development of bone morphology [13–16]. Based on the above studies, researchers assumed that the morphology of the femoral trochlea was likely to be affected by acquired factors, more than genetic predispositions.

Researches had supposed that the sliding stress of patella on the trochlear groove was the essential factor for the morphological development of femoral trochlea in the skeletal growth period after birth [17]. The lateralized patella concentrated excessive mechanical stress on the lateral condyle, which could result in the remodeling of the lateral trochlear facet. Meanwhile, the insufficient stress on the center and the medial trochlear facets could lead to the trochlear hypoplasia. As a result, the abnormal stress distribution on the trochlea might be the reason for flat trochlear groove or asymmetric trochlear facets [18]. To explore the effects of abnormal mechanical stress on the morphological development of femoral trochlea, we performed surgical patellectomy and patellar dislocation in the growing rats to change the original biomechanical stress on the trochlear groove. Up to now, this is the first study that investigates the role of mechanical stress on the development of femoral trochlea in growing rats. This study is aiming to reveal morphological changes of both bone trochlea and cartilage on the trochlea under the abnormal mechanical status. We hypothesized the prior surgical inventions could lead to a dysplastic trochlea in growing rats.

## Methods

### Study design

Thirty-six 4-week-old Wistar female rats (female, 28 days of age, weighting 40 g to 60 g) provided by the Laboratory Animal Center of Hebei Medical University were randomly assigned into three groups. The patellectomy group (PG, *n* = 12), rats in this group underwent the patellectomy. The dislocation group (DG, *n* = 12), rats in this group underwent the surgery-induced patellar dislocation. Rats in the sham group (SG, *n* = 12) underwent arthrotomy. Three weeks and twelve weeks after the surgery were chosen as the time point of detection. The rats were housed in stainless steel cages with three rats per cages at the Laboratory Animal Center of Hebei Medical University Third Affiliated Hospital. The room had a cyclical light-dark environment every 12 h and a constant temperature of 25 °C. The rats were permitted free movement in cages and free access to water and food. Rats were euthanized by excessive intraperitoneal injection of pentobarbital sodium (200 mg/kg) according to guidelines for animal euthanasia [19, 20]. All experimental protocols in this study were inspected and approved by the Animal Research Committee of Hebei Medical University Third affiliated hospital (No. Z2017–012-1).

### Surgical protocols

**Fig. 1:**
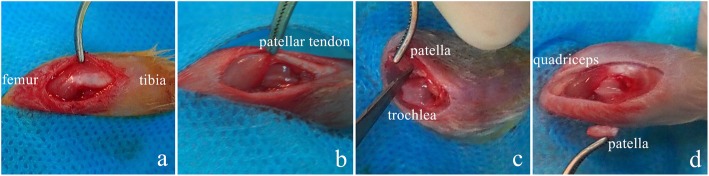
Detailed procedure of the operations: **a**, open the joint capsule through the incision of the medial retinaculum. **b**, in the dislocation group, lateral dislocating the patella using tweezers. **c**, in the patellectomy group, reversing the patellar to unfold the patellar articular facets, then separated the patellar from the tissue. **d**, in the patellectomy group, the incision was closed by 5–0 absorbable suture, in the dislocation group, the incision medial joint capsule was open for the natural healing

After three days of environmental accommodation, all of the operations were performed by an experienced team, including a senior surgeon and two operating assistants. The operative area was shaved and prepped in a sterile fashion. The rats were anesthetized with pentobarbital sodium (30 mg/kg, intraperitoneal injection). A longitudinal incision was made on the left knee joint. After separating the skin and subcutaneous tissue, the joint capsule was exposure through the medial knee approach (Fig. 1a). The patella and quadriceps tendon were reversed to unfold the articular surface of the patella in the patellectomy group. Then the patella was separated and removed from the tendinous tissue (Fig. 1c, d). Methods of surgical induced patellar dislocation had been reported in our previous studies [13, 15]. In order to dislocate the patella in this study, the medial retinaculum of the knee joint was cut off, and the patella was laterally dislocated from the trochlear groove (Fig. 1b). The state of patellar dislocation in dislocation group was maintained once the patella was dislocated due to the rupture of the medial retinaculum. The incisions were closed by 5–0 absorbable suture in the patellectomy group. Except for the skin suture, the incisions of subcutaneous tissue and joint capsule do not require the extra suture in the dislocation group. The rats received acetaminophen (30 mg/kg, daily) to control the postsurgical pain for five days.

### Safranin-O and fast green staining

Eighteen rats (PTG, *n* = 6, PDG, *n* = 6, SSG, *n* = 6) were euthanized respectively at 3 and 12 weeks after surgery. Knee specimens were soaked in 4% paraformaldehyde (pH = 7.40) overnight at 4 °C. The knees were then immersed in 10% EDTA (pH = 7.40) at 4 °C, 30 days for decalcification. A concentration gradient of ethanol and xylenes were used for dehydration, and the specimens were embedded in paraffin. Next, 5 μm sections were cut along the femoral axis to get the transverse images of the trochlear sulcus. The sections were rehydrated and stained by Safranin-O and fast green to show the cartilage and subchondral bone [21–23].

### Micro-computed tomography(μ-CT) measurement

**Fig. 2:**
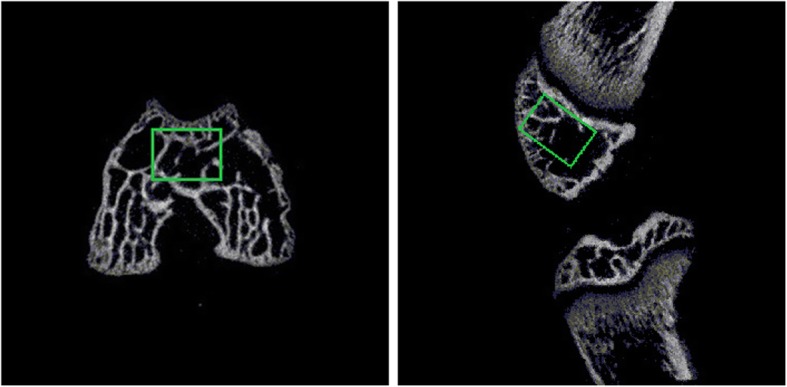
Axial and sagittal view of the distal femur. ROI was shown within the rectangular area

The knee joint was separated and immersed in 4% paraformaldehyde (pH = 7.40). A micro-computed tomography (micro-CT) scanner (Skyscan-1174, Bruker, Evere, Belgium) was used to analyze the trochlear morphology and structure of the subchondral bone. The micro-CT scanner was set at a source voltage of 50 kV, a source current of 800 μA, and a scaled image pixel size of 10 μm. Measuring methods of the femoral trochlear angle (FTA) was based on the published studies on the tomographic images which were defined by the angle between the deepest point of trochlear sulcus and peak point of the bilateral condyle. An experienced surgeon and a radiologist independently calculated the FTA, and the repetition was performed to test the intraclass correlation coefficient (ICC) with an interval of two months. Values of ICC were ranged from 0 to 1 that 0 represents no reliability, and 1 represents perfect reliability. The region of interest (ROI), an eight mm^3^ rectangular volume beneath the trochlear groove, was selected to determine the parameters of the trabecular bone (Fig. 2). Three-dimensional reconstruction of the distal femur was conducted based on the tomographic images (CTAn, N-Recon; Bruker). The following representative parameters were selected to evaluate the trabecular growth under the trochlear groove according to previous studies [24, 25]: Bone volume density (BV/TV, %), Average trabecular number (Tb.N, 1/μm), Average trabecular thickness (Tb.Th, μm), and Average trabecular separation (Tb.Sp, μm).

### Statistical analysis

Statistical analysis was performed using GraphPad Prism (GraphPad software V7, La Jolla, CA, USA). Significance was assessed using a two-way analysis of variance, and Dunnett’s multiple-comparisons was tested between each experimental group and control group at each time point. A *p*-value of less than 0.05 was considered statistically significant. All data are expressed as the mean and 95% confidence interval. According to the result of preliminary experiments, at least six rats were required at each group and each time point for a confidence level of 90% and a power of 80%(1-*β*) [22].

## Results

### Visual and histological observations

**Fig. 3:**
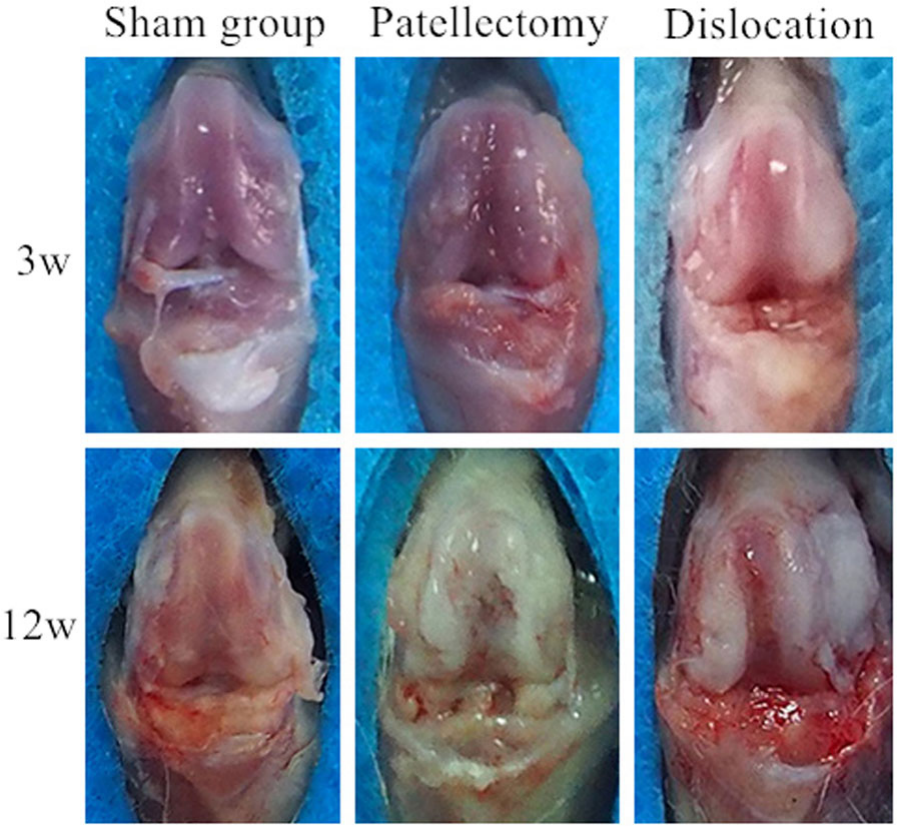
Morphology of cartilage on the trochlear groove. Normal articular cartilage of the trochlea was shown at 3 weeks in the sham group. The shape of the trochlear cartilage was blunt at 3 weeks in the PG. The cartilage on the lateral trochlear facets was thicker than the center and the medial at 3 weeks in the PG. The articular cartilage degenerated at 12 weeks in the PG. After 12-week abnormal patellar tracking by the surgical-induced dislocation, the cartilage in the center of trochlea had shown apparent degeneration and apparent proliferative tissue on the lateral trochlear facets

**Fig. 4:**
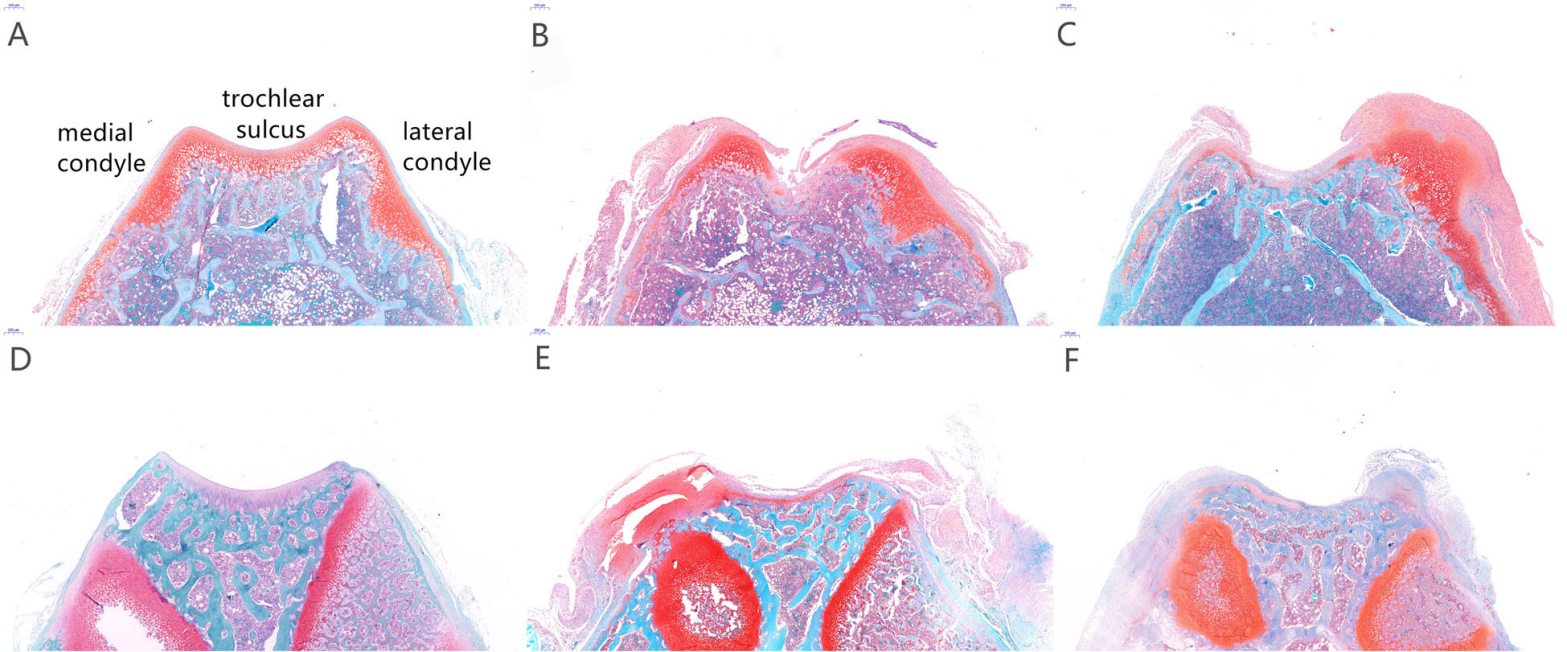
Histological findings on axial sections of femoral trochlea by Safranin-O and fast green staining. **a**,**d** Healthy articular cartilage on trochlea at 3, 12 weeks were shown in the SG. **b** In the patellectomy group, the distribution of articular cartilage was changed. The synthesis of cartilage on the bilateral condyle was increased, while decreased in the center of the trochlear groove at 3 weeks. **c** In the dislocation group, the articular cartilage on the top of the lateral trochlear facets was significantly increased at 3 weeks. **e** The PG in 12 weeks, besides the flat trochlea, the articular cartilage in the trochlear groove was visible degenerated compared with the SG. **f** In the dislocation group, the cartilage both on the trochlear center and the lateral facets was worn out, and the lateral condyle is relative convex compared with the other two groups. Cartilage tissue was red-stained, and the trabecular bone was the blue

The articular cartilage in the patellectomy group became blunt in compared with the healthy cartilage at 3 weeks. Apparent cartilage growth appeared on the lateral trochlear facets in the dislocation group at 3 weeks(Fig. 3). The trochlear groove became increasingly flat and irregular with the growth of the rats in the two experimental groups at 12 week (Fig. 3). Visual degeneration was observed in two experimental groups at 12 weeks. Under histological staining, the distribution of articular cartilage became irregular at 3 weeks compared with the sham group (Fig. 4a). In the patellectomy group, the coverage of cartilage was excessive on the top of bilateral trochlear facets, while it was decreased in the center of the trochlear groove at 3 weeks(Fig. 4b). In the dislocation group, the excessive growth of cartilage was shown on the lateral trochlear facets, while the cartilage decreased in the medial and center of the trochlear groove at 3 weeks(Fig. 4c). The degenerated changes were shown in the two experimental groups at 12 weeks in terms of the Osteoarthritis Research Society International (OARSI) classification [26]. The flat trochlea was observed in two experimental groups (Fig. 4e, f) compared with the sham group (Fig. 4d) at 12 weeks. It was worth noting that there was a noticeable bump on the lateral trochlear facets in the dislocation group at 12 weeks, which was similar to the human trochlear dysplasia (Fig. 4f).

### μCT measurement of trochlear morphology

**Fig. 5:**
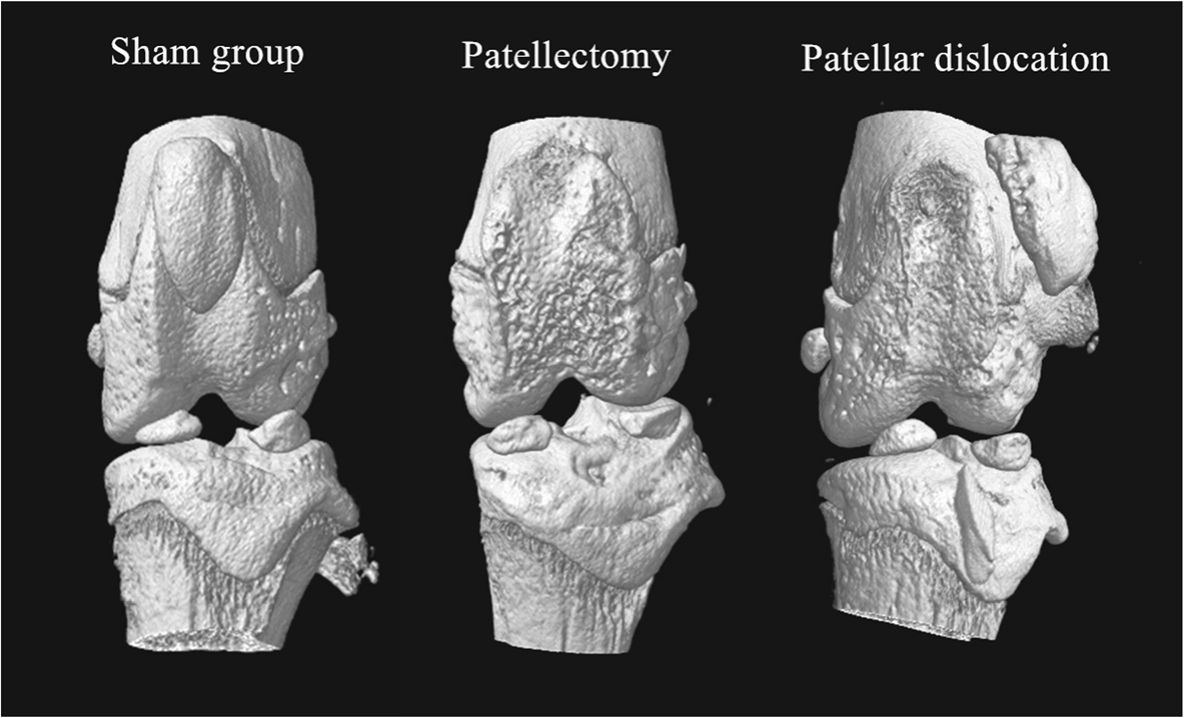
The three-dimension reconstruction of micro-CT confirmed the patellofemoral congruence of the three groups

**Fig. 6:**
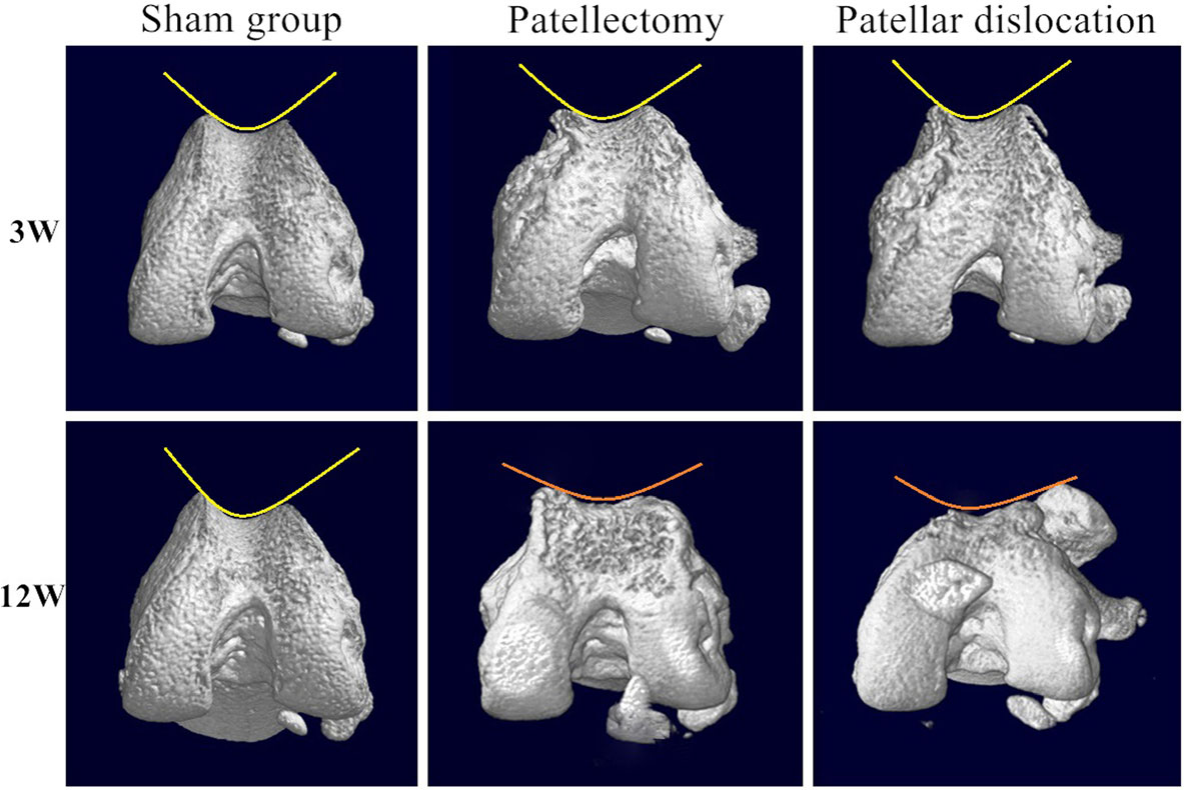
Three-dimension reconstruction of the distal femur by micro-CT scan at 3, 12 weeks. In the PG and PDG at 12 weeks, the center of the trochlear groove become flat. Compared with the flat lateral condyle in the PG, the lateral condyle in the PG remains convex under the patella tracking. No visible morphological changes on trochlea in two experimental groups at three weeks

**Fig. 7:**
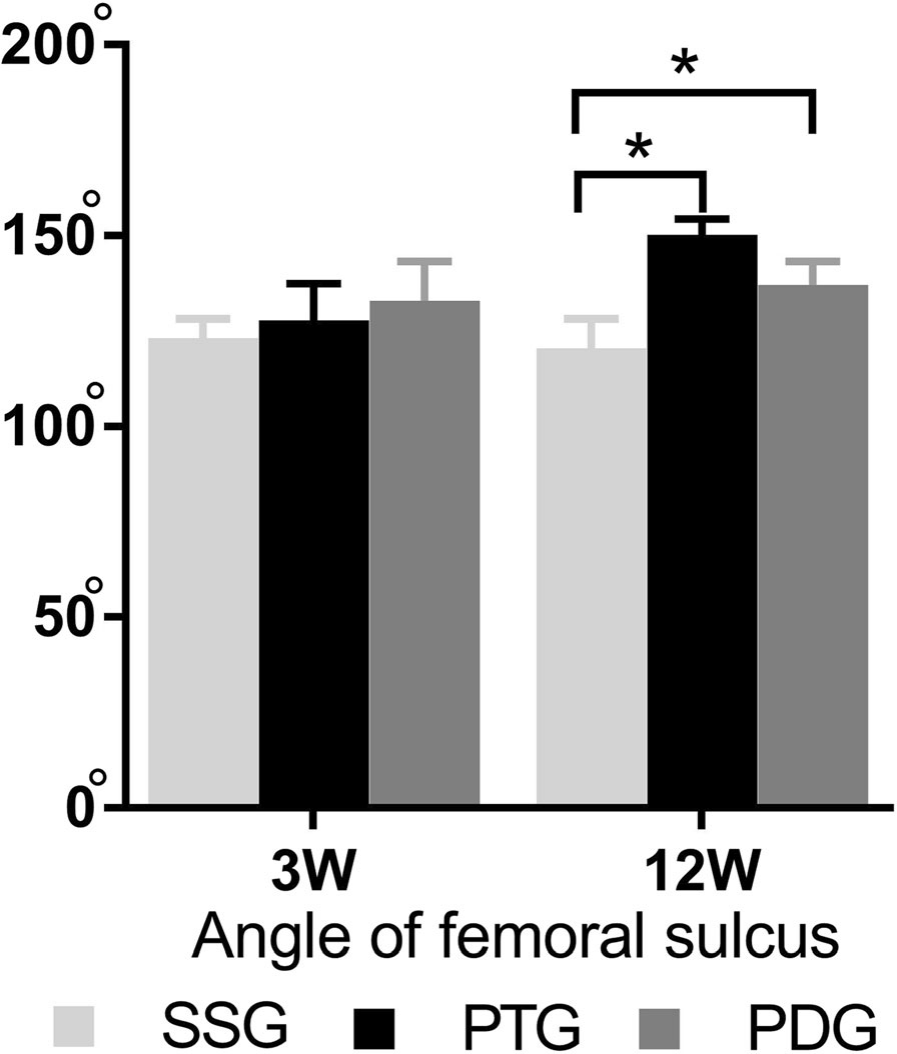
Angle of trochlear sulcus at different time point. No significant difference in the angle of femoral sulcus between experimental groups and the sham group at 3 weeks after surgery. Mean angle of femoral sulcus in each experimental groups were greater in comparison with the control group at 12 weeks, which indicated the femoral trochlea became flat. Error bars indicate 95% confidence interval. Asterisks in the graph indicate statistical significance

The three-dimensional (3D) reconstruction of the distal femur revealed the patellofemoral congruence of the three groups (Fig. 5). Moreover, the axial tomographic images were shown in Fig. 6. The intra- and inter-observer correlation coefficient were high for the femoral trochlear angle (Table 1). The mean femoral trochlear angle (FTA) was 120.9° ± 5.5° at 12 weeks in the sham group; the patellectomy group was 150.2° ± 3.1°; the dislocation group was 137.1° ± 4.4°. The FTA of two experimental groups were significantly increased than the sham group at 12 weeks (PG vs. SG P *p* = 0.001, DG vs. SG *p* = 0.005). There was no significant difference in FTA between the two experimental groups at 12 weeks(Fig. 7) (*p* = 0.06). There was no significant difference in the femoral trochlear angle in PG and DG compared with the sham group at 3 weeks (SG123.2° ± 3.6°, PG 127.8° ± 6.8°, DG 132.9° ± 7.4°, PG vs. SG *p* = 0.38, DG vs. SG *p* = 0.05) (Fig. 6).

**Table 1:** Intraclass Correlation Coefficient of Femoral Trochlear Angle(FTA)

FTA	Intra-observer	95% CI	Inter-observer	95% CI
SG(3w)	0.845	0.401-0.985	0.947	0.496-0.995
PG(3w)	0.982	0.830-0.998	0.880	0.154-0.987
DG(3w)	0.951	0.529-0.995	0.887	0.384-0.988
SG(12w)	0.874	0.211-0.987	0.747	0.427-0.974
PG(12w)	0.809	0.571-0.980	0.891	0.500-0.989
DG(12w)	0.802	0.546-0.979	0.808	0.443-0.980

*Abbreviations*: *SG* Sham group, *PG* Patellectomy group, *DG* Dislocation group, *95%CI* 95% Confidence Interval

### The quantity of subchondral trabecula under trochlear sulcus

**Fig. 8:**
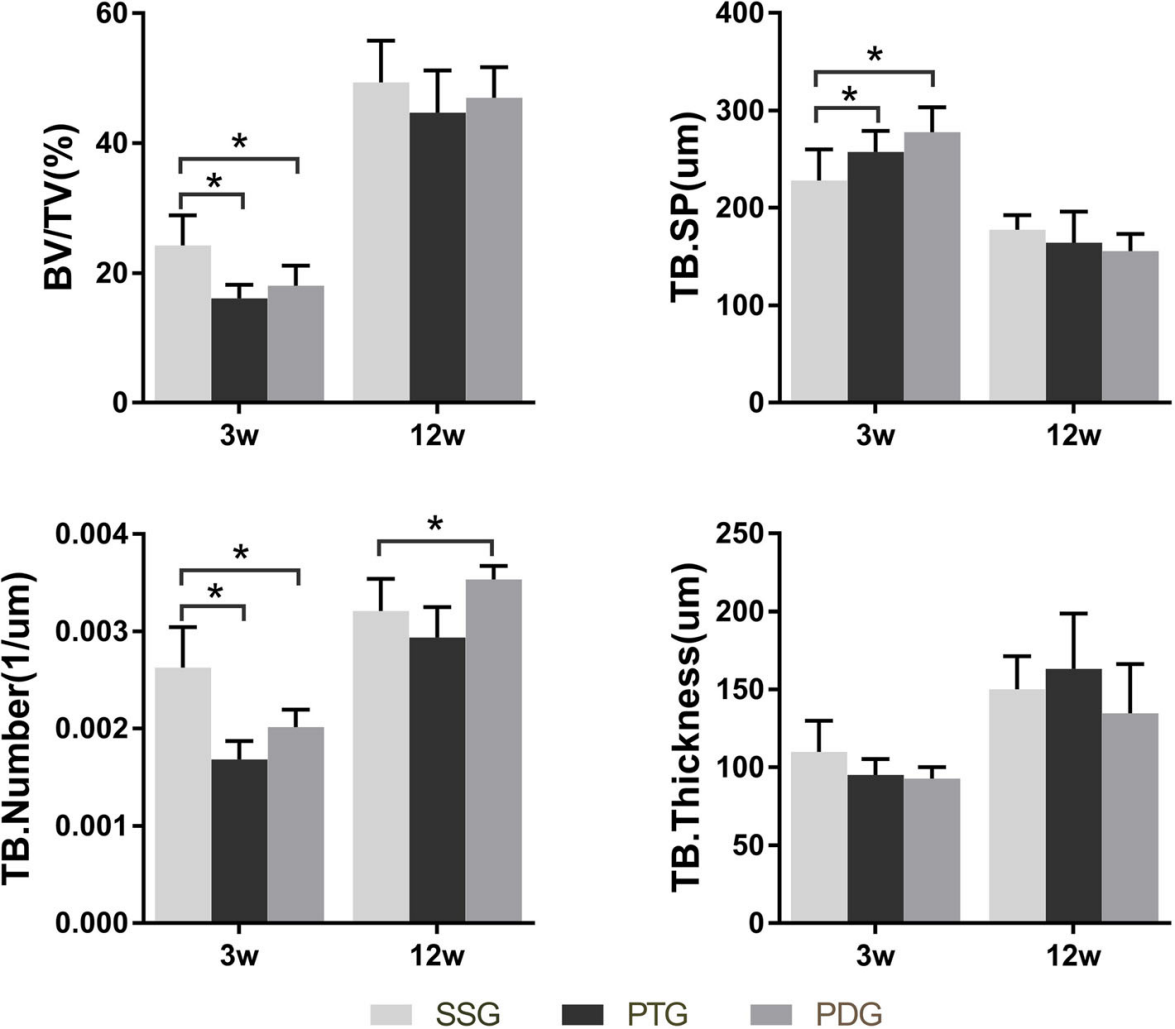
The quantity of trabecula under the trochlear groove through micro-CT analyze. The BV/TV, and TB. Number in the PG and PG were significantly decreased at 3 weeks (*P* < 0.05). The increase in TB. SP was a consistent result with the decrease of BV/TV, TB.N. The changes in quantity reflected that the interspace of trabecular was enlarged. No significant changes in TB. Thickness was found among the three groups at 3 weeks. No significant difference was found in the parameters among the three groups at 12 weeks

Compared with the healthy knee, the growth of the subchondral bone under the trochlear groove was reduced in the early stage. The average trabeculae number (Tb. N) was significantly lower in the experimental groups than the sham group at 3 weeks (SG 2.63 × 10^− 3^ ± 2.2 × 10^− 4^, PG 1.68 × 10^− 3^ ± 1.0 × 10^− 4^, DG 2.01 × 10^− 3^10^− 3^ ± 1.0 × 10^− 4^, PG vs. SG *p* = 0.001, DG vs. SG *p* = 0.002). The BV/TV and Tb. Sp is consistent with the change of Tb. N at 3 weeks. There was no significant difference between the PG and DG at 3 weeks (Fig. 8). No significant difference was found in Tb. Th among three groups at 3 weeks(SG 109.2 ± 10.9; PG 95.2 ± 5.5; DG 93.1 ± 3.9, PG vs. SG *p* = 0.28, DG vs. SG *p* = 0.20). Tb. N, BV/TV, Tb. Sp and Tb. Th did not show significant differences among the three groups at 12 weeks (Fig. 8).

## Discussion

The findings in this study proved that the morphological development of femoral trochlea was influenced by abnormal mechanical stress, which was due to the position of the patella in growing rats. In comparison with the sham group, a flat trochlear sulcus was generated by surgical induced patellectomy and patellar dislocation after 12 weeks. Besides the trochlear morphology, the coverage of articular cartilage in the center of the trochlear groove in the experimental group was reduced, and the excessive growth of cartilage was present on the border of trochlear facets where the mechanical stress was concentrated. The quantity of trabecular under the trochlea was simultaneously reduced due to the absence of sliding stress of patella at 3 weeks after the surgery.

The design of two experimental groups (patellectomy group and dislocation group) increased the reliability of experiments results. The patellectomy could decrease the mechanical stress above the trochlear groove by reducing the strength transmission between the quadriceps and patella in terms of the previous studies [27, 28]. Unlike the patellectomy, the entire extensor mechanism in the dislocation group was preserved in contrast to the complete removal of patella stress. Moreover, patellar stress was retained and transferred to the lateral side. Therefore, the different surgical methods ensured the maximum reduction of stress on the trochlear groove as well as avoiding the potential disturbance of the broken extensor mechanism. In the experiments, the 3D micro-CT reconstruction had validated the expected patellar position in the experimental groups compared with the sham group at the two points (3, 12 weeks). The similar results from the two experimental groups at 12 weeks supported that the dysplastic trochlea was resulted from the insufficient patellar stress due to the transfer of patellar position.

The causality of the development of trochlear dysplasia and the related risk factors remained unclear. However, the numerous evidence in the cross-sectional studies had proved the correlation of trochlear dysplasia and the risk factors. ØYE et al. found that nearly 45-fold higher risk of trochlear dysplasia in breech presentation than the cephalic presentation [29]. The typical effects of these abnormalities were the deviation of patellar tracking from the trochlear center and the relative insufficient stress on the trochlear groove which indicated that the role of mechanical stress in the shaping process of trochlear morphology.

Whether the morphology of the femoral trochlea would change after birth remains controversial. Studies found minor morphological changes of the dysplastic trochlea during skeletal growth and implied that the origin of TD was determined by genetic factors with human evolution [5, 12, 30]. Nevertheless, Tardieu et al. found that the trochlear groove became gradually deeper and the lateral condyle protruded because of the upright movement after birth [31]. Until now, only isolated cases have potentially supported that the morphology of femoral trochlea was affected by acquired factors. Lippacher reported an isolated patient (9-year-old) of recurrent patellar dislocation with non-operative treatments. Permanent patellar dislocation developed, and the dysplastic trochlea eventually progressed from type B to D according to the Dejour classification after four years [32]. Salzmann also presented a case of single-side flattened trochlea on a 16-year-old patient who received below-knee amputee at the age of 18 months that speculated the interaction between the biomechanical stress and the development of bone morphology [33].

In an 11-year clinical follow-up, Benoit found that the realignment surgery could remodel the dysplastic trochlea at very young ages [34]. Fu et al. also draw a similar conclusion that the morphology of trochlea improved after the patellar instability was corrected by patellar stabilization surgery in young kids after a minimum 4-year follow up [35]. A clinical study has given the femoral trochlea does not remodel after the realignment surgery in children older than ten years [36]. However, researchers in the study also found the improvement of sulcus angle younger than ten years old. Although studies have indicated the trochlear dysplasia was genetic determined, there were therapeutic studies in favor of the potential influences of developmental factors on the trochlear morphology [34–36].

For the joint instabilities, the patellofemoral joint and hip joint have structural similarities. It was well acknowledged that the position of femoral head influenced the morphology of acetabulum in the congenital hip dysplasia, which supported the role of mechanical stress on the development of the bone morphology [37–39]. During the joint development, the trochlea gradually formed a sulcus and bilateral facets to engage the patella [40]. In addition to the bone morphology, Yamada had found that the different distribution of cartilage on trochlear sulcus in patients with and without recurrent patellar dislocation [41]. Moreover, the previous studies had proved the abnormalities of trabecular under abnormal mechanical stress [42]. In this study, researchers found similar changes at different levels. The distribution of cartilage on the trochlear sulcus was remodeled at an early stage. The trabecular number was decreased at an early stage after the absence of patellar stress. The trochlear sulcus got flattened after 12 weeks of insufficient patellar stress. These findings supported that insufficient patellar stress was one of the causes affecting the morphological development of femoral trochlea after birth.

This study has limitations. First, the Wistar rat takes three months from bone development to mature [43]. Due to the limitation of surgical technics, we took the surgery in the first third period (28 days). Although we found the morphological changes, the earlier period of skeletal growth may be preferable to explore the origin of trochlear dysplasia. And untimely degeneration of articular cartilage at 12 weeks was likely to be one of the complications due to the deficiency of surgical interventions in addition to the mechanical factors. Second, in comparison with the patellar dislocation, reduction of the patella should be introduced to the experimental groups. In facts, we had reported the effects of early reduction after the patellar dislocation on the rabbit model in our previous research [15]. After that, diverse methods were carried out in the rat model to explore further pathological changes. Moreover, the reduction is now being considered in the following studies. Finally, further studies with larger specimens and diverse experiment design are required to follow up in addition to the histological and radiographic level.

## Conclusion

In conclusion, this study demonstrated that insufficient patellar stress on the femoral trochlear was a potential factor affecting the trochlear morphology in the growing stage. Surgical induced patellectomy and patellar dislocation lead to the dysplastic trochlear sulcus in growing rats. Besides the bone morphology of trochlear sulcus, the articular cartilage and subchondral trabecula under the trochlear sulcus were remodeled early stage after the surgery.

## Data Availability

The detailed data and materials of this study were available from the corresponding author through emails on reasonable request.

## Abbreviations

BV/TV: Bone volume/total volume (Bone volume density)
Micro-CT: Micro-computed tomography
ROI: Region Of Interest
Tb. N: Trabecular number (1/um)
Tb. Sp: Trabecular separation (um)
Tb. Th: Trabecular thickness (um);
TD: Trochlear dysplasia
